# The importance of data transformation in correlation analysis of factor VIII and inhibitor titers in acquired hemophilia

**DOI:** 10.1016/j.rpth.2026.103344

**Published:** 2026-01-13

**Authors:** Sergei Kulikov, Valeriya Surimova, Alexey Surenkov

**Affiliations:** National Research Centre for Haematology, Moscow, Russian Federation

Dear Editor,

In 2 recent studies of acquired hemophilia [1,2], the authors assessed the relationship between factor (F)VIII activity levels and FVIII inhibitor titers using scatter plots and linear regression/correlation analysis, concluding that no relationship exists between these variables.

However, the empirical distributions in these studies appear markedly nonnormal. Under such conditions, applying the Pearson correlation coefficient directly to raw data may yield misleading results, particularly when variables are highly skewed.

This consideration is especially relevant in acquired hemophilia, where FVIII activity and inhibitor titers often display wide variability and skewness due to heterogeneous clinical presentations. Without appropriate transformation, direct correlation analysis can obscure true biological associations.

A well-established approach to handling skewed biological data is to perform a logarithmic transformation prior to correlation analysis. This procedure helps normalize distributions, stabilize variance, and convert multiplicative relationships into additive ones, thereby better satisfying the assumptions of parametric methods, such as the Pearson correlation.

To illustrate this point, we analyzed data from 76 people with newly diagnosed acquired hemophilia treated at the National Research Centre for Haematology (Moscow, Russia) between 2018 and 2025.

**Figure 1:**
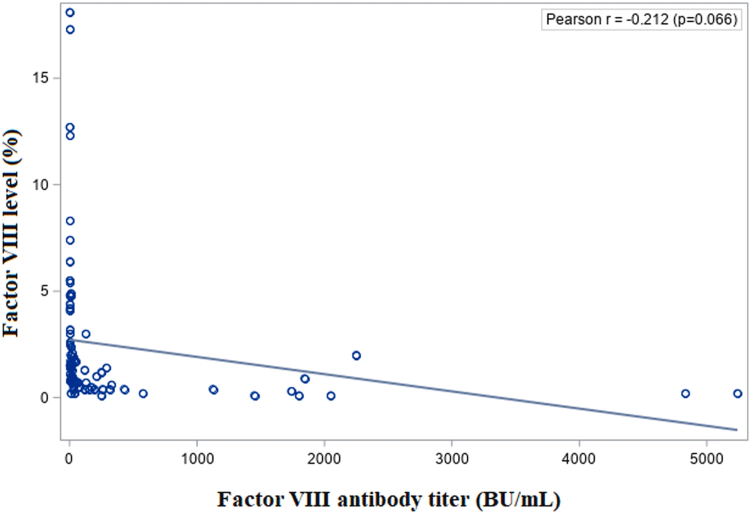
Simple regression of factor (F)VIII activity vs FVIII inhibitor titers (raw data). BU, Bethesda Units.

Analysis of the raw FVIII activity and inhibitor titers revealed a weak, nonsignificant correlation (r = −.21; *P* = .066; Figure 1), consistent with the nonnormality confirmed by Kolmogorov–Smirnov tests (*P* = .01 for both parameters). In such cases, it is appropriate to apply the Spearman correlation, which yielded r = −.76 and *P* < .0001. This demonstrated a significant association. However, for a more robust evaluation and clearer visualization, we recommend applying a logarithmic transformation.

**Figure 2:**
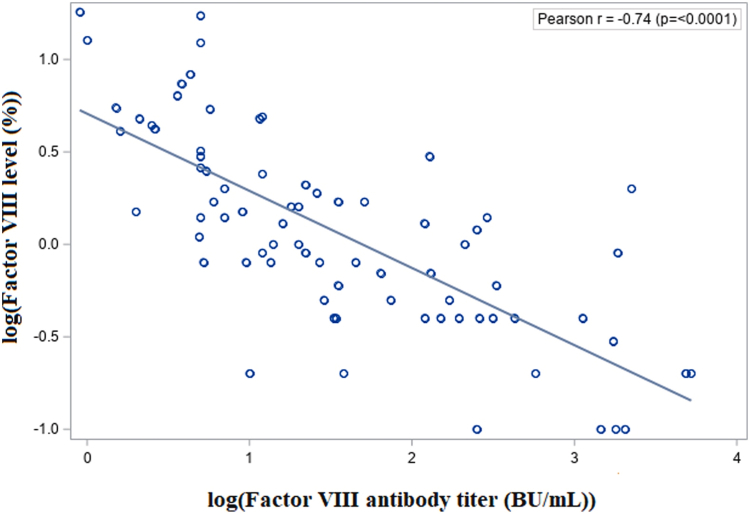
Simple regression of log_10_(factor [F]VIII activity) vs log(FVIII inhibitor titers). BU, Bethesda Units.

After logarithmic transformation of both variables, their distributions approached normality (*P* > .15 for FVIII; *P* = .045 for inhibitor titers), and a strong, highly significant negative correlation emerged (r = −.74; *P* < .0001; Figure 2).

These findings demonstrate that neglecting appropriate data transformation can lead to erroneous conclusions, specifically, the false inference of no correlation when a strong linear relationship exists in a log-transformed space.
